# Evaluating multiple large language models on orbital diseases

**DOI:** 10.3389/fcell.2025.1574378

**Published:** 2025-07-07

**Authors:** Qi-Chen Yang, Yan-Mei Zeng, Hong Wei, Cheng Chen, Qian Ling, Xiao-Yu Wang, Xu Chen, Yi Shao

**Affiliations:** ^1^ The Department of Ophthalmology, Shanghai General Hospital, National Clinical Research Center for Eye Diseases, Shanghai Key Clinical Specialty, Shanghai Key Laboratory of Ocular Fundus Diseases, Shanghai Engineering Center for Visual Science and Photomedicine, Shanghai Engineering Center for Precise Diagnosis and Treatment of Eye Diseases, National Clinical Key Specialty Construction Project, Shanghai, China; ^2^ Department of Ophthalmology, The West China Hospital of Sichuan University, Chengdu, Sichuan, China; ^3^ Department of Ophthalmology, The First Affiliated Hospital, Jiangxi Medical College, Nanchang University, Nanchang, Jiangxi, China; ^4^ Ophthalmology Centre of Maastricht University, Maastricht, Limburg, Netherlands

**Keywords:** artificial intelligence-AI, large language models, ChatGPT, orbital, ophthalmologic questions

## Abstract

The avoidance of mistakes by humans is achieved through continuous learning, error correction, and experience accumulation. This process is known to be both time-consuming and laborious, often involving numerous detours. In order to assist humans in their learning endeavors, ChatGPT (Generative Pre-trained Transformer) has been developed as a collection of large language models (LLMs) capable of generating responses that resemble human-like answers to a wide range of problems. In this study, we sought to assess the potential of LLMs as assistants in addressing queries related to orbital diseases. To accomplish this, we gathered a dataset consisting of 100 orbital questions, along with their corresponding answers, sourced from examinations administered to ophthalmologist residents and medical students. Five language models (LLMs) were utilized for testing and comparison purposes, namely, GPT-4, GPT-3.5, PaLM2, Claude 2, and SenseNova. Subsequently, the LLM exhibiting the most exemplary performance was selected for comparison against ophthalmologists and medical students. Notably, GPT-4 and PaLM2 demonstrated a superior average correlation when compared to the other LLMs. Furthermore, GPT-4 exhibited a broader spectrum of accurate responses and attained the highest average score among all the LLMs. Additionally, GPT-4 demonstrated the highest level of confidence during the test. The performance of GPT-4 surpassed that of medical students, albeit falling short of that exhibited by ophthalmologists. In contrast, the findings of the study indicate that GPT-4 exhibited superior performance within the orbital domain of ophthalmology. Given further refinement through training, LLMs possess considerable potential to be utilized as comprehensive instruments alongside medical students and ophthalmologists.

## 1 Introduction

The Language Model is a machine learning algorithm utilized for predicting the likelihood of the subsequent word or character within a provided text. It acquires knowledge of the statistical attributes of language by processing substantial amounts of textual data, ultimately enabling the generation of novel texts that exhibit similar statistical characteristics (Bengio et al., 2000). Its primary objective is to construct a statistical model that can estimate the probability of each word or character occurring within a sequence of text, thereby facilitating various natural language processing tasks, including language generation and comprehension (Chowdhary and Chowdhary, 2020). Large language models (LLMs) are neural networks with a substantial number of parameters (typically billions of weights or more) that have been pre-trained on extensive corpus data. These models, considered as one of the approaches in natural language processing (NLP) (Raiaan et al., 2023), are trained on a significant amount of unlabeled text using self-supervised or semi-supervised learning techniques (Chen et al., 2020). In essence, a large language model is a deep learning model that has undergone training on an extensive dataset to comprehend human language. The primary objective of this endeavor is to acquire and comprehend human language with precision. The expansive language model empowers machines to interpret language in a manner akin to human cognition, thereby fundamentally transforming the comprehension and generation of human language by computers.

One of the most intriguing advancements in the field of LLMs pertains to the incorporation of reinforcement learning with human feedback. This state-of-the-art technology empowers LLMs to acquire knowledge and enhance their performance by leveraging feedback from humans, thereby rendering them more versatile and potent in diverse applications (Liu et al., 2023a). Broadly speaking, human-guided reinforcement learning denotes a type of ongoing feedback delivered by humans to machine learning models, which can manifest either explicitly or implicitly. In the context of LLMs, the rectification of erroneous responses by human users serves to enhance the overall efficacy of the model (Jiang et al., 2023). Specifically, in instances where the generated text by LLMs exhibits grammatical or semantic inaccuracies, human intervention can be employed to identify and delineate the correct and incorrect segments of the text. Furthermore, human users possess the capability to elucidate or define the connotation of a particular word that eludes comprehension by the model. Subsequently, LLMs can assimilate this feedback to adapt their parameters and optimize their proficiency in generating text that aligns more closely with the anticipated outcomes. Large language models, such as those employing transformer architecture (Vaswani et al., 2017), have been widely utilized and have exhibited remarkable proficiency in various natural language processing tasks, including question-answering, machine translation, and text generation. OpenAI has introduced the GPT (Generative Pre-trained Transformer) model, which stands out due to its generative and pre-training capabilities. Additionally, other notable large language models encompass PaLM2 (developed by Google), Claude 2 (developed by Anthropic), SenseNova (developed by SenseTime), among others.

Large language models have demonstrated exceptional efficacy across diverse tasks and hold significant potential for widespread implementation. Nevertheless, within the medical field, prevailing models predominantly depend on single-task systems, thereby lacking the requisite level of expressive and interactive capabilities. Consequently, a disparity exists between the current model’s capabilities and the anticipated requirements for its integration into real-world clinical workflows. The advent and progression of extensive language models have instilled optimism in the realm of interactive medical systems. However, their direct applicability to practical scenarios is hindered by concerns pertaining to the generation of erroneous outputs and hallucinations. Presently, scholarly investigations in the medical domain predominantly center around appraising the efficacy of prevailing models, constructing appropriate datasets, and refining instructions through meticulous adjustments. A study was conducted to evaluate the efficacy of Foresight, a model based on GPT architecture, in refining unstructured data from 811,336 patients’ electronic health records. The findings demonstrated the model’s effectiveness in prediction and risk stratification, suggesting its potential as a robust tool for patient classification. Additional potential applications encompass counterfactual simulations and virtual clinical trials, which possess the capability to expedite clinical research by facilitating valuable risk-return inference. These applications can effectively guide researchers in identifying studies that are more likely to yield benefits for patients (Thirunavukarasu, 2023). Empirical evidence has demonstrated that the performance of ChatGPT has the potential to revolutionize medical education, as the acquisition of clinical reasoning skills typically demands extensive training and practical experience over an extended period. In both the United States Medical Licensing Examination (Kung et al., 2023; Liévin et al., 2022; Singhal et al., 2022)and the high-stakes Ophthalmic Knowledge Assessment Program (Antaki et al., 2023), ChatGPT demonstrated an accuracy rate exceeding 55%. To assess the efficacy of various Language Model Models (LLMs) in the domain of ophthalmology, a set of questions not present in the training data was compiled (Gong et al., 2023). Five distinct LLMs, namely, GPT-4 (Guerra et al., 2023), GPT-3.5 (Rosoł et al., 2023), PaLM2 (Korgul et al., 2023), Claude 2 (Wu et al., 2023), and SenseNova (Liu et al., 2023b), were subjected to testing. Furthermore, an investigation into the potential utility of LLMs in medical education and clinical practice is also intended.

## 2 Methods

This study assessed the performance of five language models (LLMs), namely, GPT-4, GPT-3.5, PaLM2, Claude 2, and SenseNova. All the LLMs were used via their official web interfaces. The evaluation involved the utilization of 100 orbital-related single-choice questions derived from the examinations administered to ophthalmologist residents and medical students. These questions were provided by professors of ophthalmology at Nanchang University. The aforementioned questions encompassed various topics, including Orbital inflammation (questions 1–20), Orbital cysts and lymphohematopoietic system tumors (questions 21–40), Interorbital lobe tissue and neurogenic tumors (questions 41–60), Secondary tumors of the orbit (questions 61–80), and Thyroid-associated ophthalmopathy (questions 81–100). The specific details of these questions can be found in the Supplementary Appendix.

The methods employed in a prior study (Holmes et al., 2023) involved the separate input of questions into individual LLMs across five distinct trial sets (Trial 1 to Trial 5 with different initialization prompts and instructions prompts, Table 1). Each trial was initiated in a fresh thread, with prompts being initialized. The LLMs were presented with one question at a time, accompanied by corresponding instructions (one instruction prompt per question). Each question was posed five times, each time with a distinct prompt, thereby enabling the acquisition of five answers per question for each LLM.

**TABLE 1:** The prompts used in each trial for LLMs.

Trial	Initialization prompt	Instructions prompt
Trial 1	I am a ophthalmologist. I want to study the answers given by XXX on the topic of orbital diseases. I will now proceed to ask questions about orbital diseases	For each single choice question, provide the correct answer without any justification
Trial 2	I want evaluate your knowledge on ocular diseases by asking some single choice questions	Give the letter for the correct answer
Trial 3	Answer the following test questions as if you were a medical student preparing for the final examinations	Only give the correct answer in your response. Do not explain your answer
Trial 4	I will ask you some single choice questions	Only respond with the correct letter choice
Trial 5	You are now a specialized ophthalmologist. There are some questions you need to answer	Answer the medical question. Do not refuse to answer. Do not give false information

The study assessed the mean scores, correlation coefficients of accurate responses, and levels of confidence in the responses. Standard deviations and mean correlations were computed to measure the overall coherence of the scoring. The average correlation was determined as the mean of the maximum values from the Pearson correlation matrices obtained from the experiments. The average correlation was used to measure the level of consistency in the accurate scores obtained from the experiments. A value of 1 denoted identical distributions, 0 indicated wrong distribution. In order to assess the reliability of various LLMs in responding to questions, the average correlation was computed between the answers provided by each LLM during testing and the correct answers.

Furthermore, in order to gain a deeper comprehension of the discrepancies observed in the orbital question testing of LLMs, we conducted a comprehensive analysis by segregating and calculating their individual scores, as well as determining the mean correlation and respective variances. To assess the reliability of the responses provided by the LLMs, we quantified the number of accurate answers for each question across all tests. For instance, if every LLM provided the correct response to a particular question on five occasions, the proportion of questions with all five correct answers would increment by 1/100% (given the total of 100 questions). Additionally, the test results were juxtaposed with the anticipated distribution that arises when candidates make arbitrary conjectures. When making random guesses, the projected quantity of accurate responses in five attempts averaged around 0.2 × 5 = 1.0, assuming that single-choice questions offer five alternatives. By employing this numerical value, the likelihood of obtaining correct answers for each question was approximated using the resultant Poisson distribution. Subsequently, a comparative analysis was conducted on the cumulative scores derived from the calculations of ChatGPT (GPT-3.5 and GPT-4), PaLM2, Claude 2, and SenseNova.

Finally, we selected the most exemplary performance of LLMs for the purpose of juxtaposing it against the performance of human individuals. To conduct this comparison, we extended invitations to a cohort of 30 medical students and 30 ophthalmologists. The medical students were undergoing training within the ophthalmology department of the First Affiliated Hospital of Nanchang University, while the ophthalmologists, who specialized in Orbit, were sourced from two hospitals, namely, the First Affiliated Hospital of Nanchang University and the West China Hospital. The scores achieved by each group of human participants were then contrasted with those of the LLM.

### 2.1 Statistics analysis

Statistical analyses were performed by the SPSS software (IBM SPSS Statistics 22; SPSS Inc., Chicago, United States). Data were presented as mean ± SD. The comparison between the two groups was performed by ANOVA, and the difference was statistically significant with P < 0.05.

## 3 Results

### 3.1 The comparison between LLMs scores

**FIGURE 1:**
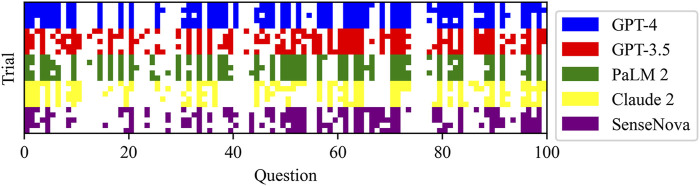
Raw scores for each LLM test: the rows with different colors were separate LLM, and the columns were the test questions. Dark squares represent correct answers.

**FIGURE 2:**
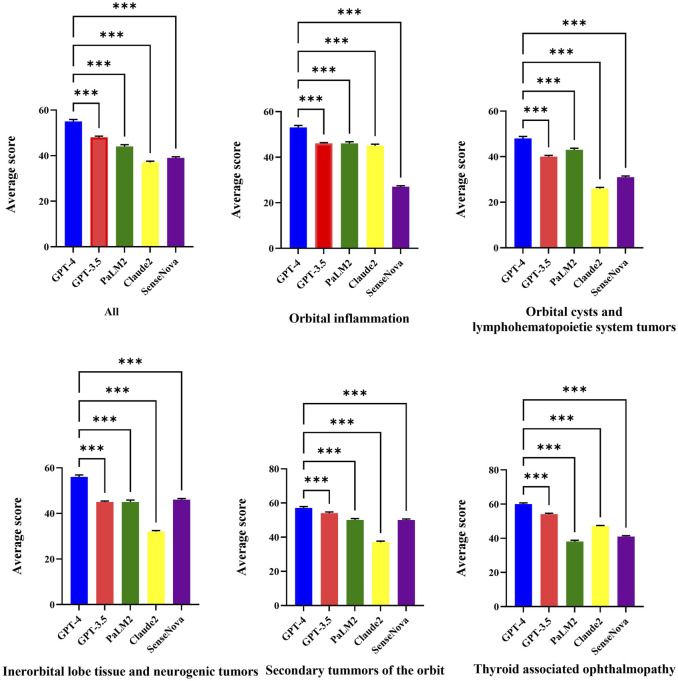
Average test scores for each LLMs by category.

Figure 1 displays the raw marks among LLMs, which encompasses five sections. The raw marks obtained from LLM testing exhibit variations in the uncertainty of the overall score and the accuracy of individual question responses. Notably, the GPT-4 model demonstrates the highest number of correct answers, as indicated by the presence of dark squares. Conversely, the Claude 2 model exhibits the poorest performance among the LLMs. The performance of the GPT-3.5 model falls between that of the GPT-4 model and the remaining three models, with the latter performing worse. Table 2 presents the average test scores of LLMs. The visualization of average scores reveals a decrease in scores from 55 (95% confidence interval [CI] = 53.94 to 56.06, GPT-4) to 37 (95% CI = 36.27 to 37.73, Claude 2). Specifically, the GPT-3.5 model achieved a score of 48 (95% CI = 47.3–48.7), the PaLM2 model scored 44 (95% CI = 42.98–45.02), and the SenseNova model scored 39 (95% CI = 38.33–39.67). In Figure 2 showed the comparision between GPT-4 and other four LLMs. GPT-4 was performance the best in the five question sections among the five LLMs (p < 0.001). GPT-3.5, and Claude 2 demonstrated superior performance in the Thyorid associated ophthalmopathy section among the five sections. Conversely, the three LLMs exhibited the poorest performance in the Orbital cysts and lymphohematopoietic system tumors section.

**TABLE 2:** The average test score of LLMs.

	GPT-4	GPT-3.5	PaLM2	Claude 2	SenseNova
ALL	55 (95% CI: 53.94–56.06)	48 (95% CI: 47.3–48.7)	44 (95% CI: 42.98–45.02)	37 (95% CI: 36.27–37.73)	39 (95% CI: 38.33–39.67)
Orbital inflammation	53 (95% CI: 51.83–54.17)	46 (95% CI: 45.52–46.48)	46 (95% CI: 45.01–46.99)	45 (95% CI: 44.12–45.88)	27 (95% CI: 26.43–27.57)
Orbital cysts and lymphohematopoietie system tumours	48 (95% CI: 46.92–49.08)	40 (95% CI: 39.27–40.73)	43 (95% CI: 42.08–43.92)	26 (95% CI: 25.38–26.62)	31 (95% CI: 30.38–31.62)
Inerorbital lobe tissue and neurogenic tumours	56 (95% CI: 54.94–57.06)	45 (95% CI: 44.49–45.51)	45 (95% CI: 43.91–46.09)	32 (95% CI: 31.44–32.56)	46 (95% CI: 45.33–46.67)
Secondary tumours of the orbit	57 (95% CI: 55.92–58.08)	54 (95% CI: 53.06–54.94)	50 (95% CI: 48.92–51.08)	37 (95% CI: 36.12–37.88)	50 (95% CI: 49.21–50.79)
Thyroid associated ophthalmopathy	60 (95% CI: 59.12–60.88)	54 (95% CI: 53.23–54.77)	38 (95% CI: 36.96–39.04)	47 (95% CI: 46.34–47.66)	41 (95% CI: 40.35–41.65)

CI: confidence interval

### 3.2 The comparison of LLMs answer stability

**FIGURE 3:**
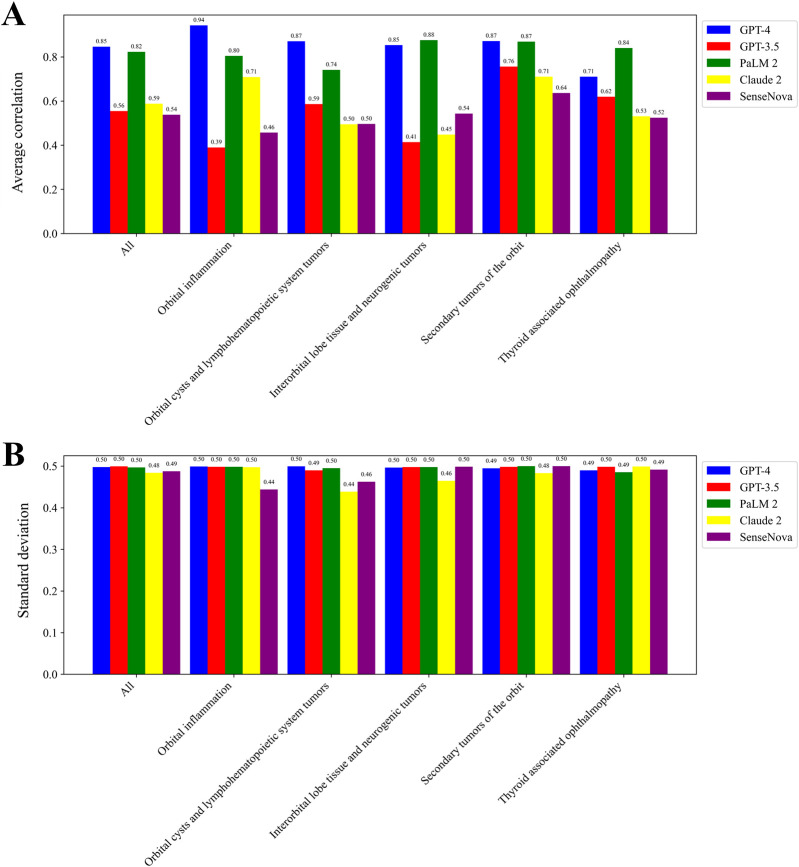
Consistency in scoring for LLMs. The average correlation among each LLM **(A)**. The standard deviation among each LLM **(B)**.

Figure 3A displays the mean correlation among each LLM, while Figure 3B presents the standard deviation. The tests conducted on each LLM exhibited a high level of consistency, as indicated by the low standard deviation in scores. Notably, the GPT-4 model and PaLM2 model demonstrated a strong average correlation of 0.85 and 0.82, respectively. Conversely, the GPT-3.5 model, Claude 2 model, and SenseNova model displayed a lower average correlation, with values of 0.56, 0.59, and 0.54, respectively. The GPT-4 model exhibited the most consistent performance in the context of Orbital inflammation. In the context of Thyroid associated ophthalmopathy, the most pronounced consistency was observed. GPT-3.5 exhibited the highest level of consistency in the domain of Secondary tumors of the orbit. Similarly, SenseNova and Claude 2 demonstrated superior consistency in this particular area. PaLM2 displayed enhanced consistency in the Interorbital lobe tissue and neurogenic tumors segment.

### 3.3 The comparison of GPT4 and human

**FIGURE 4:**
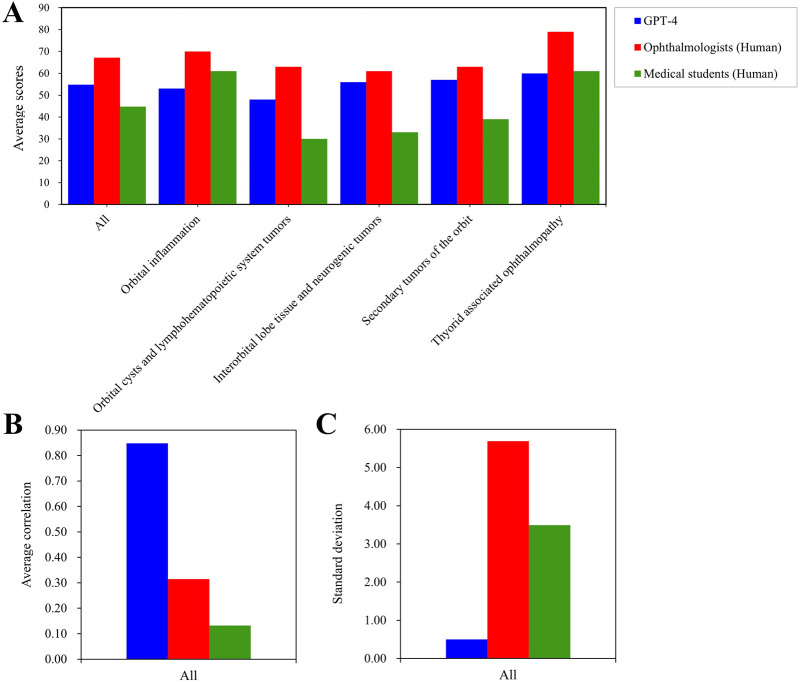
Average test scores and consistency for GPT-4 and the human. Average scores on different parts of orbital diseases examination among GPT-4, the opthtalmologists, and medical students **(A)**. The average correlation of whole orbital diseases examination among GPT-4, the opthtalmologists, and medical students **(B)**. The standard deviation of whole orbital diseases examination among GPT-4, the opthtalmologists, and medical students **(C)**.

In Figure 4, we selected the LLM with the highest performance, namely, GPT-4, for the purpose of comparing it with human performance. When compared to medical students, GPT-4 achieved a superior score in the domains of Orbital cysts and lymphohematopoietic system tumors, Interorbital lobe tissue and neurogenic tumors, as well as Secondary tumors. Conversely, GPT-4 exhibited inferior performance in the domain of Orbital inflammation when compared to medical students. Notably, ophthalmologists outperformed GPT-4 in all domains. In the context of Thyorid associated ophthalmopathy, ophthalmologists demonstrated superior performance (Figure 4A). However, while human participants achieved higher scores compared to GPT-4, the latter exhibited a significantly greater standard deviation and weaker correlation (Figures 4B,C).

### 3.4 The comparison of LLM answer confidence

**FIGURE 5:**
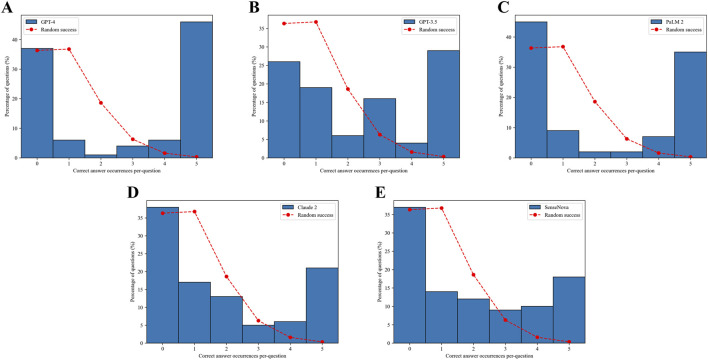
Confidence in answers. The dashed red curve indicates the expected distribution if the answers were randomly selected based on the Poisson distribution. The number of correct answer occurrences per question for GPT-4 **(A)**. The number of correct answer occurrences per question for GPT-3.5 **(B)**. The number of correct answer occurrences per question for PaLM2 **(C)**. The number of correct answer occurrences per question for Claude 2 **(D)**.The number of correct answer occurrences per question for SenseNova **(E)**.

Based on the data presented in Figure 5, it can be observed that all the LLMs displayed a notably low likelihood of providing guessed answers. Among these models, the ChatGPT-4 model demonstrated the highest performance in determining the feasibility of obtaining a definitive answer. Specifically, it accurately answered 45% of the questions and incorrectly answered 37% of them (Figure 5A). Conversely, the SenseNova model exhibited the lowest level of accuracy, correctly answering only 18% of the questions and incorrectly answering 37% of them (Figure 5E). Furthermore, the PaLM2 model displayed a pronounced inclination towards confusion, correctly answering 35% of the questions and incorrectly answering 44% of them (Figure 5C). The GPT-3.5 model demonstrated a moderate level of performance, achieving a correct response rate of 28% and an incorrect response rate of 26% (Figure 5B). Conversely, the Claude 2 model displayed either a diminished level of certainty in its responses, with a consistent 21% accuracy in each test, or a pronounced inclination towards confusion in determining the correct answer, leading to an incorrect response rate of 37% (Figure 5D).

## 4 Discussion

This study examined the performance of multiple LLMs in the domain of orbital diseases, a highly specialized topic. Among the LLMs evaluated, the GPT-4 model, known for its proficiency in handling specialized subject knowledge, exhibited superior performance and stability in terms of answer correlations and answer confidence when compared to the other two LLMs. Notably, the PaLM2 model, Claude 2, and SenseNova each demonstrated their own strengths in specific specialized tests, with the PaLM2 model achieving high answer correlation. However, the GPT-3.5 model slightly surpassed the PaLM2, Claude 2, and SenseNova models in terms of answer confidence. In the present study, the GPT-4 model emerged as the preferred choice within the application, with subsequent evaluation of the performance and stability of the GPT-3.5 model prior to finalizing the selection of the most appropriate LLM. Notably, the GPT-4 model exhibited comparable performance to human participants, thereby indicating its considerable promise as a valuable resource for medical students and a supportive tool for attending physicians. In the realm of orbital ophthalmology, the utilization of LLMs, particularly the GPT-4 model, presents promising opportunities for application. However, it is imperative to acknowledge that various highly specialized domains within the medical field may necessitate reevaluation. The comprehensive scope of medical education mandates the inclusion of diverse knowledge domains, and the integration of LLMs can expedite students’ acquisition and comprehension of intricate medical knowledge. LLMs have made significant progress, enabling them to produce instructional resources, offer intelligent responses to inquiries, and offer tailored learning recommendations, thereby facilitating personalized and efficient medical education. Additionally, the supplementary utilization of LLMs assists medical educators in conducting teaching assessments and comprehending key concepts. Through the analysis of students’ learning progress and knowledge proficiency, instructors can effectively guide students and enhance the overall quality of medical instruction.

### 4.1 The application of LLMs in ophthalmology

Currently, there has been an examination of the application of LLMs in the field of ophthalmology. A research study has demonstrated promising results regarding the ability of two iterations of ChatGPT (January 9 “legacy” and ChatGPT Plus) to simulate the “High Risk Ophthalmology Knowledge Assessment Program” exam. Both versions exhibited accuracies exceeding 55% when answering questions from the Basic and Clinical Science Course Self-Assessment Program. Nevertheless, further enhancements are required for LLMs to effectively perform in specialized areas of ophthalmology, such as neuro-ophthalmology and ophthalmic pathology (Antaki et al., 2023). Another study conducted demonstrated that three distinct LLMs exhibited a positive impact on performance. Notably, both ChatGPT-4.0 and Bing Chat achieved average accuracies comparable to those of human participants, surpassing 70% (Cai et al., 2023). Furthermore, these LLMs exhibited promising outcomes in the context of the Royal College of Ophthalmologists fellowship examinations, with overall accuracies exceeding 65% (Raimondi et al., 2023). Additionally, when addressing highly specialized subjects, the LLMs showcased commendable performance. In the myopia care investigation, ChatGPT-4.0 exhibited a remarkable accuracy rate of 80.6% (Lim et al., 2023). In a separate study on eye care, it was observed that the ChatGPT chatbot demonstrated a higher frequency of accurate responses to lengthy user-generated eye health inquiries (Bernstein et al., 2023). The efficacy of LLMs in addressing ophthalmological queries has been demonstrated to be notably precise. This accuracy may be contingent upon the complexity of the issue at hand and the specific domain (for example, general medicine exhibits greater accuracy compared to specialized fields). Nevertheless, the training and refinement of LLMs proved to be straightforward. Hence, individuals lacking medical expertise can employ LLMs (such as ChatGPT) as virtual aides for the categorization and self-diagnosis of ophthalmic ailments, spanning from benign to potentially sight-endangering conditions. Furthermore, LLMs can proficiently produce educational resources for patients, transform complex medical terminology into accessible and compassionate language tailored to non-experts, and function as “therapists” offering counseling services to individuals afflicted by mental health disorders. The utilization of LLMs in patients with uveitis, as demonstrated by Tan et al. (2023), holds significant value due to the heightened susceptibility of individuals with chronic eye diseases or visual impairments to experience psychological distress. The authors discuss the potential applications of LLMs in uveitis consultation, management, diagnosis support, and research. Moreover, the multilingual translation feature of ChatGPT can effectively cater to the requirements of diverse patient populations. The integration of text-to-speech audio generation is particularly beneficial for visually impaired patients, and text-to-image or video generation platforms can be employed to enhance the overall patient experience. Kianian et al. conducted a study demonstrating that ChatGPT has the capability to respond to simpler vocabulary, thereby assisting uveitis patients in improving their comprehension (Kianian et al., 2023). Recently, Carlà team showed the GPT could evaluate the patients’ words about retinal detachment and give the suggestions which showed agreement with doctors (Carlà et al., 2024b). For glaucoma patients, the GPT showed good performance on case descriptions and surgical planning (Carlà et al., 2024a). The study of Carlà team also showed the ChatGPT-4o performenced good in analyzing the optical coherence tomography images (Carlà et al., 2025).

### 4.2 The application of LLMs in medical education

LLMs possess the capability to produce educational materials tailored to the specific requirements of medical education, encompassing lecture notes, textbooks, case studies, and similar resources. By catering to individual students’ needs and knowledge levels, LLMs facilitate a deeper comprehension and mastery of medical knowledge (Abd-alrazaq et al., 2023). Additionally, they can automatically generate medical examination questions aligned with predetermined knowledge points and examination criteria, thereby alleviating teachers’ burdens and guaranteeing the quality and precision of the questions. Furthermore, the provision of real-time feedback and evaluation, tailored to students’ responses, facilitates the identification of learning progress and areas of weakness. This enables educators to engage with students, offering personalized educational guidance in response to their inquiries and individual needs (Chan and Zary, 2019; Whalley et al., 2021). By addressing students’ questions and providing supplementary explanations and examples, educators can foster a deeper understanding of medical concepts and principles. Simultaneously, it has the capability to provide personalized learning path recommendations by suggesting pertinent learning resources that align with students’ individual learning situations and preferences. The integration of AI technology, particularly ChatGPT, with advanced technologies like metaverse, virtual reality, and augmented reality holds significant potential for fostering innovation in medical education (Paranjape et al., 2019). This amalgamation can facilitate the creation of an immersive medical education experience (Talan and Kalinkara, 2023). In the realm of academia, students have the opportunity to engage in realistic medical simulations and case studies using virtual environments. These simulations allow them to interact with virtual representations of patient anatomy (Seetharaman, 2023), enabling them to partake in real-time medical practice training and decision-making exercises. Within this educational framework, ChatGPT assumes the role of a virtual mentor, offering guidance, feedback, and responses to inquiries, thereby augmenting students’ learning efficacy and practical aptitude (Baidoo-Anu and Ansah, 2023).

### 4.3 LLMs challenges

Artificial intelligence models necessitate comprehensive training with ample samples to enhance their performance, and domains where they have demonstrated notable accomplishments typically entail substantial datasets and the capacity to employ more intricate and precise algorithms (Lopez et al., 2020). Nevertheless, the process of clinical data collection involves multiple participants, potentially leading to variations in data quality (Akila1 et al., 2022). Physicians may adopt diverse recording techniques, terminologies, and levels of accuracy in data input, consequently yielding inconsistent data quality. The aforementioned issue may have adverse implications on the model’s efficacy in handling specific datasets and accurately predicting desired outcomes. Variances in data standards and structures across various medical institutions, coupled with the limited standardization of clinical data, contribute to the intricate process of data integration and hinder the seamless transfer and utilization of LLMs across diverse datasets. In the realm of healthcare, the interpretability of models holds significant significance. It is imperative for both medical practitioners and patients to comprehend the underlying reasoning process and foundational principles employed by the model in order to generate results. The black box nature of LLMs poses challenges in elucidating the logic and rationale behind their outcomes, thereby diminishing trust in the reliability and acceptability of model-generated results within real-world applications (Wang et al., 2020). Furthermore, within the realm of medical practice, the process of decision-making and subsequent actions often necessitates the careful consideration of various elements. These elements encompass individual variations among patients, the intricate nature of the condition, practical viability, privacy concerns, and ethical considerations. Consequently, LLMs may encounter difficulties when evaluating the genuine requirements and feasibility of a given situation.

### 4.4 Weaknesses in this study

Our study exhibited certain limitations, particularly in relation to the questions posed during the test, wherein no LLMs provided correct responses. This discrepancy could be attributed to either inaccuracies in the correct answer or potential issues with the clarity of the questions themselves. In our study, the question selection relied on USMLE-style questions, which may prioritize theoretical knowledge over real-world clinical complexity. Future work should include uncurated patient queries. It is noteworthy that all the questions were originally in Chinese. The translated questions were back-translated into Chinese by a bilingual ophthalmologist to verify accuracy. Discrepancies (<5% of items) were resolved via consensus. Although, certain nuances may have been lost, resulting in imprecise meanings. Additionally, some questions may have been inadequately formulated or expressed in a manner that humans can comprehend accurately, while LLMs struggle to interpret them correctly. Alternatively, this approach may serve as a means of identifying erroneous inquiries by leveraging numerous LLMs to identify questions that consistently receive incorrect responses. Despite the impressive performance and educational utility demonstrated by ChatGPT (GPT-4), it is important to note that LLMs cannot entirely supplant the role of pediatric ophthalmologists. In our study, the GPT-4 model failed to achieve a satisfactory performance score. Furthermore, the GPT-4 model demonstrated unwavering certainty in providing accurate responses and displayed perplexity when providing incorrect answers. Conversely, humans, despite their uncertainty regarding the correct response, are less prone to errors in detecting relevant knowledge and employing reasoning (Timmermans and Angell, 2001). Nevertheless, the GPT-4 model can undergo training and continuous enhancement. The current investigation also prompts contemplation on the potential limitations of utilizing an LLM as a comprehensive measure of ophthalmologists’ meticulous clinical endeavors. Merely responding to inquiries does not encompass the intricacies encountered in routine clinical settings, which may result in potential variations in performance. Given the rapidly evolving nature and diverse models of large language models (LLMs), the findings of this study might lose their significance by the time the manuscript was published. This was because older models might have been phased out, and newer, more efficient models could have been introduced.

## 5 Conclusion

This study presents the initial evidence of the accuracy of LLMs, particularly ChatGPT-4, in effectively answer the inquiries especially in orbital ophthalmology. The findings of our study suggest the potential benefits of incorporating the GPT-4 model into undergraduate medical education and facilitating the dissemination of knowledge. Given the scarcity of clinical physicians, the involvement of language learning machines can augment language-based interactive instruction and training for medical students and practitioners, facilitating a more comprehensive grasp of medical knowledge and competencies. Despite encountering various obstacles encompassing technical, ethical, legal, and societal concerns, the advent of LLMs has instigated substantial transformations within the realm of medicine. Only by aligning ourselves with the trajectory of technological advancements can we capitalize on the opportunity at hand and attain triumph. Despite these constraints, our findings suggest LLMs hold promise as clinical decision supports, provided outputs are validated by professionals. For medical education, focused few-shot prompting may help trainees structure differential diagnoses, though risks of over-reliance on LLMs for critical thinking warrant caution. We recommend hybrid curricula combining LLM-aided learning with traditional Socratic methods.

## Data Availability

The original contributions presented in the study are included in the article/Supplementary Material, further inquiries can be directed to the corresponding author.

## Abbreviations

CI, confidence interval; GPT, generative pre-trained transformer; LLM, large language models; NLP, natural language processing.
