# Correction: Evaluating multiple large language models on orbital diseases

**DOI:** 10.3389/fcell.2025.1664098

**Published:** 2025-08-13

**Authors:** Qi-Chen Yang, Yan-Mei Zeng, Hong Wei, Cheng Chen, Qian Ling, Xiao-Yu Wang, Xu Chen, Yi Shao

**Affiliations:** ^1^ The Department of Ophthalmology, Shanghai General Hospital, National Clinical Research Center for Eye Diseases, Shanghai Key Clinical Specialty, Shanghai Key Laboratory of Ocular Fundus Diseases, Shanghai Engineering Center for Visual Science and Photomedicine, Shanghai Engineering Center for Precise Diagnosis and Treatment of Eye Diseases, National Clinical Key Specialty Construction Project, Shanghai, China; ^2^ Department of Ophthalmology, The West China Hospital of Sichuan University, Chengdu, Sichuan, China; ^3^ Department of Ophthalmology, The First Affiliated Hospital, Jiangxi Medical College, Nanchang University, Nanchang, Jiangxi, China; ^4^ Ophthalmology Centre of Maastricht University, Maastricht, Limburg, Netherlands

**Keywords:** artificial intelligence-AI, large language models, ChatGPT, orbital, ophthalmologic questions

In the published article, there was an error in **affiliation** [1]. Instead of “[Shanghai General Hospital, National Clinical Research Center for Eye Diseases, Shanghai Key Clinical Specialty, Shanghai Key Laboratory of Ocular Fundus Diseases, Shanghai Engineering Center for Visual Science and Photomedicine, Shanghai Engineering Center for Precise Diagnosis and Treatment of Eye Diseases, National Clinical Key Specialty Construction Project, Eye and ENT Hospital of Fudan University, Shanghai, China]”, it should be “[The Department of Ophthalmology, Shanghai General Hospital, National Clinical Research Center for Eye Diseases, Shanghai Key Clinical Specialty, Shanghai Key Laboratory of Ocular Fundus Diseases, Shanghai Engineering Center for Visual Science and Photomedicine, Shanghai Engineering Center for Precise Diagnosis and Treatment of Eye Diseases, National Clinical Key Specialty Construction Project, Shanghai, China]”.

The original version of this article has been updated.

