# Supplementary material for: Evaluating multiple large language models on orbital diseases
Source: Front Cell Dev Biol. 2025 Jul 7;13:1574378. doi: 10.3389/fcell.2025.1574378 (PMC12277337; doi:10.3389/fcell.2025.1574378)
Supplement: Supplementary file 1 [file DataSheet1.docx]

**Orbital-related single-choice questions**

#DECAEAEBDEDBACAECDBDADBBDADCBCECDDEAEABCDCADBDDBCADCBCCDDBECCDEBABEACCBDBBAECBECDCABEDCCCEDDDDBEBEEC# All (1-100)

1. The most important treatment for orbital cellulitis is ( )

A. Treatment of primary lesions

B. Active surgery

C. Appropriate corticosteroid therapy

D. Adequate broad-spectrum antibiotics

E. Local strengthening of anti-inflammatory effects with eye drops and eye ointments

2. The serious complications of orbital cellulitis are ( )

A. Diplopia

B. Eyeball protrusion

C. Optic nerve atrophy

D. Orbital abscess

E. Infection spreading intracranial

3. What is correct about orbital cellulitis ( )

A. Divided into two types: anterior or posterior to the orbital septum

B. The most common cause is facial swelling

C. Can cause Cavernous sinus thrombophlebitis

D. Pupil abnormalities are mainly caused by the involvement of ciliary nerve nodes

E. It is a specific inflammation

4. The main causes of orbital cellulitis are ( )

A. Sinus teeth infection

B. Edge furuncle and swelling

C. Orbital trauma

D. Orbital Periostitis

E. Orbital surgery

5.The correct explanation for the onset of orbital cellulitis is ( )

A. The most common pathogens are Staphylococcus aureus and Pseudomonas aeruginosa

B. Adults are more prone to this than children

C. Most of them are derived from hematogenous metastasis of systemic infectious diseases

D. Often accompanied by penetrating eye injuries

E. Due to the absence of valves in the orbital, facial, and sinus veins, which communicate with each other, infections in adjacent tissues are easily spread to the orbit through veins

6. What is correct for orbital cellulitis ( )

A. It may be accompanied by Keratitis

B. Multiple absences of bulbar conjunctival edema

C. Pathogens first invade adjacent Arteriole

D. Generally, it does not affect vision

E. There are acute and chronic symptoms

7. The manifestations of orbital abscess do not include ( )

A. High fever

B. Eyeball protrusion

C. Deep orbital pain

D.Photophobia

E. Visual impairment in the early stage

8. The most common symptoms of orbital periosteal abscess ( )

A. Visual impairment

B. Eye displacement

C. Eyelid edema

D. Eye movement disorders

E. Intracranial infection

9. The description of orbital Osteomyelitis is incorrect ( )

A. Residual foreign bodies after trauma can cause

B. Caused by the spread of infection in adjacent tissues

C. Bloodborne infection pathway

D.The common site is the inferior orbital wall

E. Common bacteria are Streptococcus

10. The complications of ocular Fasciitis are ( )

A. Eye perforation

B. Endophthalmitis

C. Severe damage to visual function

D. Intraorbital abscess

E. Select All Above

11. Regarding the etiology and clinical signs of orbital inflammatory pseudotumor, the error is ( )

A. It is chronic non-specific inflammation

B. Belonging to autoimmune diseases

C. Painless exophthalmos

D. Upper eyelid retraction and delayed upper eyelid drop

E. Visual impairment

12. The correct treatment for inflammatory pseudotumors is ( )

A. The most sensitive to corticosteroids is basophilic granulosa

B.The most sensitive type to radiotherapy is lymphocyte infiltration

C. Generally, local medication has good effects

D. If necessary, complete surgical resection should be performed to prevent recurrence

E. The dosage of corticosteroids should be small and short to avoid systemic complications

13. What is correct about inflammatory pseudotumors ( )

A. Edema at the attachment point of the extraocular muscles

B. Extraocular muscle hypertrophy without involvement of tendons

C. The Inferior rectus muscle was the earliest affected

D. Mostly chronic inflammation

E. The treatment is mainly based on broad-spectrum antibiotics

14. The characteristics of inflammatory pseudotumors do not include ( )

A. It can mainly affect a single tissue in the orbit, and can also extensively affect soft tissue in the orbit; ; ; ;

B. Adults suffer from monocular diseases, while children suffer from binocular diseases

C. Fibroplastic inflammation presents more severely

D. When the lacrimal gland is involved, there may be swelling above the outer orbit, congestion and edema of the eyelid conjunctiva

E. When the eye muscles are affected, the tendons also become thicker and swollen

15. Inflammatory pseudotumors are common in ( )

A. Chronic proliferative inflammation

B. Atypical cell proliferation

C. Benign cell proliferation

D. Acute inflammatory hyperplasia

E. Malignant cell proliferation

16. The CT manifestations of orbital inflammatory pseudotumor myositis are as follows ( )

A. Thickening of one or more extraocular muscles

B. Superior rectus muscle and Medial rectus muscle were mostly involved

C. Diffuse hypertrophy of the entire eye muscle

D. The edges are often blurry

E. Neat edges

17. What is the incorrect statement about painful ophthalmoplegia ( )

A. It is idiopathic inflammation of Cavernous sinus with headache and ophthalmoplegia

B. Young adults are common

C. No disappearance of light reaction

D. Accompanying nausea and vomiting

E. Give Corticosteroid treatment

18. The typical clinical manifestations of painful ophthalmoplegia include ( )

A. Headache

B. Nausea and vomiting

C. Unilateral ophthalmoplegia

D. Bilateral ophthalmoplegia

E. Monocular diplopia

19. For Wegener Granuloma, the right thing is ( )

A. The most common areas are the throat and trachea

B. The characteristic lesions are necrotic Granuloma and small Vasculitis

C. Granuloma is formed by necrotic tissue, epithelioid cells and multinucleated giant cells

D. Organs such as lungs and kidneys are not affected

E. The mucosal surface is covered by complete pseudo Stratified columnar epithelium, and the interstitial is loose and edematous

20. Which of the following statements about Wegener Granuloma is correct ( )

A. The typical triad is upper and lower respiratory tract and Sinusitis

B. Necrotizing Granuloma inflammation and Vasculitis with moderate vascular lesions

C. P-ANCA has a higher positive rate

D. The most common chest X-ray features are infiltrating lesions and nodular shadows in the lungs, which may be accompanied by local atelectasis

E. Clinical renal damage is rare

21. Dermoid cysts originate from ( )

A. Epidermal ectoderm during embryonic stage

B. Embryonic mesoderm

C.Embryonic epidermal mesoderm

D. Embryonic neuroectoderm

E. Cranial nerves Spinal Cells

22. Dermoid cysts are best found in ( )

A. Inner superior orbital margin

B. Inner inferior orbital margin

C. Outer inferior orbital margin

D. Outer superior orbital margin

E. Intramuscular conus

23. What is the correct description of cavernous hemangioma ( )

A. The capsule is often incomplete

B. The tumor contains smooth muscles

C. Often affects vision

D. The internal reflection of A-ultrasound is medium to low wave

E.Most can spontaneously subside

24. Benign tumor with orbital mass adhesion at the bone suture, usually should be considered ( )

A. Dermoid tumor

B. Dermoid cyst

C. Papilloma

D. Nerve Fibroma

E. Orbital osteoma

25. The most common orbital Benign tumor in children is ( )

A: Capillary hemangioma

B: Cavernous hemangioma

C: Rhabdomyosarcoma

D: Dermoid cyst

E: Optic Glioma

26. The most common orbital Benign tumor in adults is ( )

A. Cavernous hemangioma

B. Meningioma

C. Dermoid cyst

D. Rhabdomyosarcoma

E. Inflammatory pseudotumor

27. Cavernous hemangioma is a type of ( )

A. Vascular proliferative tumor

B. Vascular varicose tumor

C. Vascular Dilatoma

D. Vascular hamartoma

E. Arteriovenous fistula

28. Intraorbital cavernous hemangioma, the most characteristic CT manifestation is ( )

A. The tumor is located within the muscle cone

B. The tumor enhancement scan showed uneven enhancement in the early stage

C. Significant tumor enhancement

D. Dense nodular shadows within the tumor

E. Tumor adjacent to eyeball

29. What is the correct description of cavernous hemangioma ( )

A. The capsule is often incomplete

B. The tumor contains smooth muscles

C.Often affects vision

D. The internal reflection of A-ultrasound is medium to low wave

E. Most can spontaneously recover

30. Which of the following is correct about orbital hemangioma ( )

A. Most tumors are composed of fibrous tissue

B. No obvious pathological changes in the orbit can exclude the presence of lesions

C.The presence of intraorbital venous stones can confirm intraorbital hemangiomas

D.X-ray examination has localization, quantification, and qualitative value in the diagnosis of hemangioma

E. Tumor body without capsule

31. Which of the following statements is incorrect about orbital Lymphatic vessel ( )

A. More common in children under the age of 10

B. Most cases have a slow course of disease

C. A few cases may suddenly worsen due to intraorbital bleeding

D. Recurrence is easy after surgical resection

E. Due to the lack of specific drug treatment, all patients should undergo surgical resection or preoperative biopsy for a clear diagnosis

32. The correct description of orbital varicose veins is ( )

A. Mostly traumatic

B. Less congenital

C. Appearing as intermittent or postural exophthalmos, with prolonged course of disease resulting in sunken eyeballs

D. Commonly seen on the right side

E. Fatty accumulation and protruding eyeballs in patients with a longer course of disease

33. Orbital lesions with postural exophthalmos ( )

A. Cavernous hemangioma

B. Frontal ethmoid sinus mucocele

C. Orbital abscess

D. Orbital varicose veins

E. Lymphoma

34. Orbital tissue pulsation accompanied by noise, seen in ( )

A. Orbital varicose veins

B. Nerve Fibroma

C. Meningoencephalocele

D. Carotid Cavernous sinus fistula

E. Optic Glioma

35. Orbital pulsation without a murmur ( )

A. Cavernous hemangioma

B. Carotid Cavernous sinus fistula

C. Orbital abscess

D. Orbital varicose veins

E. Meningoencephalocele

36. Eye signs are commonly seen in the internal carotid artery Cavernous sinus fistula ( )

A. Pulsatile exophthalmos

B. Eyelid ptosis on the same side

C. Inability of bilateral eyeball cohesion

D. Optic papilla edema

E. Hypoesthesia of the forehead

37. The clinical manifestations of Burkitt's lymphoma, which is not orbital lymphoma, are as follows ( )

A. Diplopia

B. Upper eyelid ptosis

C. Eye movement disorders

D. Delayed or absent pupil reflex to light

E. Exophthalmos

38. The clinical characteristics of orbital mucosa-associated lymphoid tissue lymphoma are incorrect ( )

A. Common eyeball depression

B. Eyelid swelling

C. May have conjunctival edema

D. May have eye movement disorders

E. May have diplopia and decreased vision

39. The wrong description of diffuse large B-cell lymphoma is ( )

A. Belonging to B-cell lymphoma

B. Low malignancy

C.Sensitivity to chemotherapy

D. Likely to occur in the elderly

E. The most common non-Hodgkin lymphoma in adults

40. Leukemia patient, 18 years old, with 1.5cm visible in the left orbit × 2. Ocm × A 2.0cm sized lump with protruding eyeballs. The most likely type of leukemia for this patient is ( )

A. Acute mono-leukemia

B. Acute lymphocytic leukemia

C. Acute myeloid leukemia

D. Chronic lymphocytic leukemia

E. None of the above

41. The most common primary malignant tumor in the orbit in children is ( )

A. Dermoid cyst

B. Epidermoid cyst

C. Malignant meningioma

D. Rhabdomyosarcoma

E. Retinoblastoma

42. Which pathological type of Rhabdomyosarcoma has the best prognosis ( )

A. Embryonic type

B. Small bubble leaf type

C. Polymorphism

D. Epithelial type

E. Mesenchymal type

43. Rhabdomyosarcoma is common in ( )

A. Upper nasal quadrant

B. Lower nasal quadrant

C. Within the muscular cone

D. Subtemporal quadrant

E. Superior temporal quadrant

44. 75% of Rhabdomyosarcoma occurs before several years of age ( )

A. 7 years old

B. 8 years old

C. 9 years old

D. 10 years old

E. 11 years old

45. Rhabdomyosarcoma is the first choice for treatment ( )

A. Enucleation of orbital contents

B. Radiotherapy+chemotherapy

C. Chemotherapy

D. Surgery+chemotherapy

E. Surgery+radiotherapy

46. If Rhabdomyosarcoma is confined to the orbit, it does not accumulate ( )

A. Over 75%

B. More than 80%

C. More than 85%

D. More than 90%

E. Over 95%

47. The wrong description of Rhabdomyosarcoma is ( )

A. It is the most common primary orbital malignant tumor in children

B. There may be ptosis of the upper eyelid

C. The tumor has not yet invaded the orbital bone wall and is limited to the orbit, resulting in a higher survival rate after treatment

D. Orbital enucleation is the preferred treatment

E. Likely to occur in the upper part of the orbit

48. According to the Benign tumor of the orbit ( )

A. Vascular sarcoma

B. Rhabdomyoma

C. Lymphoma

D. Mesothelioma

E. Chondrosarcoma

49. The clinical characteristics of optic Glioma are wrong ( )

A. It is a benign or low-grade malignant tumor caused by the abnormal proliferation of Glia in the optic nerve

B. It is mostly seen in children, and some patients are accompanied by Neurofibromatosis

C. Originating from the orbit, it can cause visual impairment in the early stage and easily spread to the intracranial area

D. CT can display spindle-shaped enlargement of the optic nerve

E. Frequent enlargement of the optic canal with intracranial spread

50. The correct description of optic Glioma is ( )

A. Most of them are well-differentiated astrocytomas

B. Mostly oligodendroglioma

C. Primary intracranial lesions have less invasiveness

D. Multiple posterior visual pathways

E. Originating from the orbital optic nerve, which is mostly spherical in shape

51. Which item is wrong about optic Glioma ( )

A. The initial onset age is mostly 2-6 years old

B. Slow progress

C. Edema or atrophy of the optic disc

D. No significant changes in vision

E. CT shows spindle-shaped enlargement of the optic nerve

52. The wrong description of optic Glioma is ( )

A. Early visual impairment

B. Papilledema

C. Late visual impairment

D. More common in preschool children

E. Nystagmus may occur

53. Male patient, 9 years old, has chronic painless exophthalmos in the right eye with visual acuity of 0.02. Ultrasound shows spindle shaped enlargement of the optic nerve with clear boundaries and lack of internal echoes. The most likely diagnosis is ( )

A. Retinoblastoma

B. Optic Glioma

C. Optic nerve meningioma

D. Striated muscle

E. Cavernous hemangioma

54. The common lesions of the optic nerve sheath are ( )

A. Glioma

B. Neuroschwannoma

C. Meningioma

D. Inflammation

E. Nerve Fibroma

55.The CT signs of meningioma that are not optic nerve sheath are ( )

A. Calcification is common

B: Enhance the appearance of a "track" sign

C. A few can be accompanied by Neurofibromatosis

D. The optic canal can expand

E. Optic nerve thickening

56. What is correct about the description of optic nerve sheath meningioma ( )

A. Multiple bilateral occurrence

B. Rare calcification within the tumor

C. Growth along the epidural space

D. Penetrating the dura mater and growing outward centrifugally

E. Centripetal growth through the dura mater

57. The typical tetralogy of optic nerve sheath meningioma does not include ( )

A. Eyeball protrusion

B. Loss of vision

C. Chronic Papilledema

D. Intracranial metastasis

E. Visual ciliary short circuit angiogenesis

58. Regarding optic nerve sheath meningioma, the error is ( )

A. Painless and slow decline in vision

B. Sudden changes in the condition can be accompanied by intraorbital bleeding

C. Having optic nerve atrophy and abnormal blood vessels in the optic papilla

D. CT shows a spindlel swelling at the apex of the orbit

E. Commonly seen in middle-aged women

59. Which of the following statements is incorrect regarding the clinical characteristics of optic nerve sheath meningioma ( )

A. More common among middle-aged women

B. Painless

C. Papilledema can be seen in fundus

D. CT examination can reveal diffuse or spherical enlargement of the optic nerve

E. Prone to malignant transformation

60. The following statement is incorrect ( )

A. Cavernous hemangioma belongs to benign orbital mesodermal tumors

B. Rhabdomyosarcoma is the most common malignant orbital mesodermal tumor in children

C. CT scan of optic nerve meningioma often presents with spindle shaped enlargement of the optic nerve

D. Rupture of a dermoid cyst may cause orbital cellulitis

E. Orbital green tumor is a light green mass formed by limited infiltration of leukemia cells under the periosteum of the orbital bone, which develops rapidly

61. Secondary tumors in the orbit usually originate from primary tumors in which body part ( )

A. Stomach

B. Breast

C. Lung

D. Kidney

E. Liver

62. Orbital metastatic tumors refer to ( )

A. Orbital malignant tumor metastasis to bone

B. Orbital malignant tumors metastasize to the lungs

C. Orbital malignant tumors metastasize to the sinuses

D. Malignant tumors in other parts metastasize to the orbit

E. Orbital malignant tumor metastasis to intracranial area

63. What is the most typical clinical manifestation of orbital Retinoblastoma ( )

A. Blurred vision

B. Eyeball protrusion

C. Visual defects in color

D. Eyelid swelling

E. White pupil syndrome

64. The highest incidence rate of malignant intraocular tumors in children is ( )

A. Uvea Melanoma

B. Retinoblastoma

C. Intraocular metastatic tumors

D. Retinal hemangioma

E. Lymphoma

65. The most common age group of orbital Retinoblastoma is ( )

A. 0-5 years old

B. 6-10 years old

C. 11-15 years old

D. 16-20 years old

E. 21-25 years old

66. The current treatment of Retinoblastoma is wrong ( )

A. To improve the quality of life of children, early enucleation is generally no longer advocated

B. chemotherapy is only used for advanced palliative treatment

C. When removing the eyeball, the removal of the optic nerve should be greater than 10mm

D. scleral surface radiation therapy is superior to external radiation therapy

E. If the tumor is larger than 50% of the eyeball volume, vitreous seeding and vision loss, Enucleation of the eye should still be performed, and postoperative adjuvant radiotherapy and chemotherapy should be performed

67. The growth mode of Retinoblastoma includes ( )

A. Endogeneity

B. homogeneity

C. multifocal

D. diffuse infiltrative

E. above are all

68. The characteristic of Retinoblastoma is that ( )

A. mostly hereditary

B. inheritance has an Rb gene mutation, which is located in the 4th band of chromosome 13 long arm region 1

C. may be Asymptomatic or have strabismus and white pupil

D.The typical B-ultrasound change is "calcium plaque"

E. late stage can lead to secondary glaucoma, exposed Keratitis and systemic metastasis

69. The following description of Retinoblastoma is wrong ( )

A. The tumor boundary is relatively clear

B. The most common malignant tumor in the eyeball of infants and young children

C. It is a malignant tumor of nerve Endoderm

D. Tumors may grow intracranial

E. Intratumoral calcification is a characteristic manifestation

70. The following statement about Retinoblastoma is wrong ( )

A. In younger patients, bilateral Retinoblastoma is more common than unilateral

B. long-term survival rate of unilateral patients is higher than bilateral

C. the second non-ocular tumor only occurs in chemotherapy patients

D. unilateral Retinoblastoma is more common than bilateral

E. the most common malignant tumor in children

71. Which of the following genes is abnormal in patients with Retinoblastoma ( )

A. NF-1

B. RB1

C. APC

D. p53

E. Bcl-2

72. Female, 2-year-old, with protruding eyes. CT shows a soft tissue mass in the left eyeball with patchy calcification and thickened optic nerve. The most likely diagnosis is ( )

A. Melanoma

B. Inflammatory pseudotumor

C. Cavernous hemangioma

D. Retinoblastoma

E. Rhabdomyosarcoma

73. The correct description of malignant Melanoma is ( )

A. More common among children and young people

B. The appearance of the tumor is black, but it can also be brown or light red

C. The prognosis of tumors located near the corneal margin is worse than that of tumors located in the lacrimal caruncle

D. When the thickness of the tumor is greater than 2mm, metastasis is less likely to occur

E. Melanocytes are not sensitive to freezing, and surgery is the first choice

74. At present, the wrong thing about the treatment of Choroid malignant Melanoma is ( )

A. The tumor has a small volume, a thickness of 1mm, and a diameter of 3mm, which can be observed regularly

B. The tumor is located in the posterior pole, with a thickness of 7mm and a diameter of 15mm, and laser photocoagulation is feasible

C. The tumor is located at the equator, with a thickness of 5mm and a diameter of 8mm, and can be treated with radiation therapy

D. The tumor is located in the surrounding area, with a thickness of 2mm and a diameter of 7mm, and can be locally removed

E. Tumor orbital metastasis, feasible orbital content enucleation surgery

75. For patients with Basal-cell carcinoma of the eyelid who do not involve the orbit and eyeball, the preferred treatment is ( )

A. Simple radiotherapy

B. Simple surgery

C. Surgery+postoperative radiotherapy

D. Surgery+postoperative chemotherapy

E. Surgery+postoperative synchronous radiotherapy and chemotherapy

76. Meibomian gland carcinoma invading the orbit is prone to occur ( )

A. No transfer occurs

B. Direct spread

C. Planting transfer

D. Lymphatic metastasis

E. Hematological metastasis

77. Which of the following orbital diseases cause the eyeball to shift outward ( )

A. Thyroid associated ophthalmopathy

B. Malignant tumor of lacrimal gland

C. Frontal sinus mucocele

D. Squamous cell carcinoma of Maxillary sinus

E. Optic nerve sheath meningioma

78. Orbital ultrasound shows medial orbital lesions, which are circular, extrapyramidal, with clear and smooth boundaries, and low internal reflection. The following lesions are most likely to be diagnosed ( )

A. Orbital nerve Fibroma

B. Nasal mucocele with orbital invasion

C. Orbital metastatic carcinoma

D. Orbital pseudotumor

E. Optic nerve sheath meningioma

79. The following are not the orbital pathways for nasopharyngeal tumors ( )

A. Transsphenoidal Cavernous sinus

B. Trans Superior orbital fissure

C. Trans pterygoid process and Pterygopalatine fossa

D. Suborbital fissure entering the orbit

E. Transfrontal sinus

80. The primary site of orbital metastatic cancer in women is common ( )

A. Ovarian cancer

B. Uterine cancer

C. Breast cancer

D. Bronchial lung cancer

E. None of the above

81. What is the correct description of thyroid related ophthalmopathy ( )

A. Thyroid function examination plays an important role in the diagnosis of this disease

B. The pathogenesis is Humoral immunity

C. The lesion mainly damages the posterior orbital soft tissue and extraocular muscles

D. Diagnosis can be made based on clinical manifestations

E. Edema and hypertrophy of the entire extraocular muscle tissue

82. The description of thyroid related ophthalmopathy is incorrect ( )

A. It is the most common cause of adult exophthalmos

B. The pathological change is edema of the extraocular muscles

C. The lesions mainly damage Orbicularis oculi muscle and extraocular muscles

D. CT examination can help diagnose

E. Thyroid function examination is not very helpful for diagnosis

83. The first extraocular muscles affected by thyroid associated ophthalmopathy are ( )

A. Inferior rectus muscle muscle

B. Superior rectus muscle muscle

C. Medial rectus muscle muscle

D. Lateral rectus muscle muscle

E. Superior oblique muscle

84. The most common causes of adult exophthalmos ( )

A. Orbital cellulitis

B. Thyroid associated ophthalmopathy

C. Nerve Fibroma

D. Meningioma

E. Cavernous hemangioma

85. Excluding typical ocular manifestations of thyroid associated ophthalmopathy ( )

A. Eyelid retraction

B. Late ptosis of the upper eyelid

C. Eyeball protrusion

D. Movement disorders, diplopia

E. Spiral dilation of conjunctival vessels

86. Excluding the main treatment measures for thyroid associated ophthalmopathy ( )

A. Eye protection treatment

B. Drug anti infection treatment

C. Orbital decompression surgery

D. Optic nerve sheath incision

E. Radiation therapy

87. What is correct about thyroid related eye diseases ( )

A. Both are accompanied by hyperthyroidism

B. Both are accompanied by an increase in iodine uptake rate

C. Ophthalmic muscle abdominal hypertrophy, without involvement of tendons

D. All accompanied by ophthalmic muscle paralysis

E. If the protrusion of the eye measured by the eye protractor is greater than 28mm, it is considered a malignant protrusion

88. The description of thyroid related ophthalmopathy is incorrect ( )

A. Obvious eye symptoms

B. May be accompanied by pretibial myxedema

C. Female patients are often more likely to develop to a serious state than male patients

D. May not have symptoms of hyperthyroidism

E. Infringement of extraocular muscles and retroocular tissues

89. Which of the following is not a manifestation of thyroid related eye disease ( )

A. Most have a history of hyperthyroidism

B. Visual impairment

C. Frequent ptosis is the first symptom

D. CT shows spindle shaped hypertrophy of extraocular muscles

E. Bilateral exophthalmos

90. The treatment methods for thyroid related eye diseases mainly include ( )

A. Surgical treatment

B. Local eye protection treatment

C. External radiation therapy

D. Glucocorticoid therapy

E. All of the above are

91. The following indicators are not used to evaluate the activity of thyroid associated ophthalmopathy ( )

A. Eye MRI

B. Serum/urine marker GAGs

C. CCAS score

D. N0SPECS standard

E. Octreotide scanning

92. Thyroid associated ophthalmopathy ( )

A. Intermittent exophthalmos

B. Paranasal sinus derived exophthalmos

C. Spontaneous pulsating exophthalmos

D. Thyroid dysfunction exophthalmos

E. Thyroid dysfunction exophthalmos

93. The following are not common imaging features of thyroid associated ophthalmopathy ( )

A. Ultrasound shows irregular echoes of orbital fat and thickening of extraocular muscles

B. CT shows multiple spindle shaped hypertrophy of extraocular muscles, and compression and invagination of the ethmoid bone cardboard

C. CT shows multiple extraocular muscle hypertrophy without involving tendons

D. The image showed that the Lateral rectus muscle was thickened in one eye

E. MRI shows multiple extraocular muscle hypertrophy with increased or decreased T2 weighted imaging signal

94. What is the correct description of thyroid related non invasive exophthalmos ( )

A. The pathological changes are often unrelated to the improvement of hyperthyroidism treatment

B. Exophthalmos is caused by lesions involving the retrobulbar tissue

C. Patients often experience symptoms such as visual fatigue, foreign body sensation, fear of light, and tearing

D. Eye protrusions are usually less than 18mm

E. Frequent eye swelling, pain, diplopia, etc

95. The incorrect description of thyroid related ophthalmopathy is ( )

A. Often manifested as a certain degree of exophthalmos, accompanied by hypotropia and esotropia

B. The most common cause is abnormal thyroid function in women

C. The most frequently affected muscle is the Inferior rectus muscle

D. Patients with diplopia and abnormal head position can undergo surgical treatment when their condition stabilizes

E. The surgical method is mainly muscle weakening surgery, and muscle strengthening surgery is contraindicated

96. The following indicators are not used to determine the active phase of thyroid related eye disease ( )

A. Pain during eye movements

B. Diplopia

C. Conjunctival congestion

D. Protrusion degree

E. Late ptosis of upper eyelid

97. What is the correct description about thyroid related ophthalmopathy ( )

A. When there is thyroid related eye disease, there must be hyperthyroidism

B. The severity of hyperthyroidism may not necessarily be parallel to the degree of exophthalmos

C. Patients with hyperthyroidism must also have thyroid related eye diseases

D. When there is thyroid related eye disease, thyroid function must be abnormal

E. The severity of hyperthyroidism is opposite to the degree of exophthalmos

98. Regarding thyroid related ophthalmopathy, there are errors in the description ( )

A. The patients with thyroid associated ophthalmopathy in the advanced stage need Curative care

B. Male patients often have more severe conditions than female patients

C. Older patients are often heavier than younger patients

D. TAO patients who smoke often have more severe conditions

E. Asian patients are often more susceptible than European patients

99. Patients suspected of thyroid related ophthalmopathy often require thyroid function examination. Which of the following tests is helpful in guiding clinical diagnosis and treatment ( )

A. FT3

B. FT4

C. Highly sensitive TSH

D. Examination of thyroid antibodies

E. All of the above items

100. If the patient's thyroid function is completely normal, the most valuable signs that must be possessed for diagnosing thyroid related ophthalmopathy are ( )

A. Eyelid swelling

B. Eyeball protrusion

C. Eyelid retraction

D. Extraocular muscle hypertrophy

E. Visual impairment
